# Whole Genome Analysis of a Single Scottish Deerhound Dog Family Provides Independent Corroboration That a *SGK3* Coding Variant Leads to Hairlessness

**DOI:** 10.1534/g3.119.400885

**Published:** 2019-11-14

**Authors:** Heidi G. Parker, D. Thad Whitaker, Alexander C. Harris, Elaine A. Ostrander

**Affiliations:** Cancer Genetics and Comparative Genomics Branch, National Human Genome Research Institute, National Institutes of Health, Bethesda, Maryland

**Keywords:** *Canis lupus familiaris*, fur, hair, dog, genetics

## Abstract

The breeds of domestic dog, *Canis lupus familiaris*, display a range of coat types with variation in color, texture, length, curl, and growth pattern. One trait of interest is that of partial or full hairlessness, which is found in a small number of breeds. While the standard for some breeds, such as the Xoloitzcuintli, requires sparse hair on their extremities, others are entirely bald, including the American Hairless Terrier. We identified a small, rare family of Scottish Deerhounds in which coated parents produced a mixed litter of coated and hairless offspring. To identify the underlying variant, we performed whole genome sequencing of the dam and five offspring, comparing single nucleotide polymorphisms and small insertions/deletions against an established catalog of 91 million canine variants. Of 325 homozygous alternative alleles found in both hairless dogs, 56 displayed the expected pattern of segregation and only a single, high impact variant within a coding region was observed: a single base pair insertion in exon two of *SGK3* leading to a potential frameshift, thus verifying recently published findings. In addition, we observed that gene expression levels between coated and hairless dogs are similar, suggesting a mechanism other than non-sense mediated decay is responsible for the phenotype.

The domestic dog, *Canis lupus familiaris*, is divided into over 400 breeds which display enormous variation in morphologic phenotypes including body size, shape, tail length, leg length, etc. (Fogle 2000; American Kennel Club 2017). Within a particular breed, individuals share a large amount of both genetic and phenotypic homogeneity (Karlsson and Lindblad-Toh 2008; Parker *et al.* 2017a). This reflects bottlenecks that were critical to breed formation, including popular sires and the often limited numbers of individuals used to initiate and propagate breeding programs (Lindblad-Toh *et al.* 2005; Boyko *et al.* 2010; Parker *et al.* 2004; Ostrander *et al.* 2017). Ideally, such population structure should facilitate both linkage and association studies, however extensive linkage disequilibrium has made it difficult to move from associated or linked marker to causative mutation (Lindblad-Toh *et al.* 2005; Sutter *et al.* 2004). Whole genome sequencing (WGS) of dogs with unique phenotypes and comparison to a comprehensive catalog of variants circumvents this issue.

One of the most striking phenotypic features observed in dog is coat variation. To date, genetic studies have identified genes contolling multiple prominent coat features including curl (Salmela *et al.* 2019; Cadieu *et al.* 2009), the presence of moustache and eyebrows, also known as “furnishings” (Cadieu *et al.* 2009; Parker *et al.* 2010), shedding (Hayward *et al.* 2016), density (Wiener *et al.* 2013), presence or absence of an undercoat (Whitaker and Ostrander 2019), length (Cadieu *et al.* 2009; Housley and Venta 2006; Dierks *et al.* 2013), and, in a small number of breeds, hairlessness (O’Brien *et al.* 2005; Drögemüller *et al.* 2008; Parker *et al.* 2017b). In some cases, the same genes have proven to be important in understanding phenotypes observed in other species (Higgins *et al.* 2014; Menche *et al.* 2017; Drögemüller *et al.* 2008).

A spontaneous canine model of alopecia was recently described by Hytonen and Lohi (Hytönen and Lohi 2019) in a small family of Scottish Deerhounds, a breed which typically displays a harsh and wiry coat (American Kennel Club 2017). Simultaneously, we identified an independent Scottish Deerhound family, also segregating the hairless trait. In Deerhounds, the disorder mimics patterns observed in some children with hair growth early in childhood. Dogs are born with fur but regress to complete alopecia by two to three months of age, yet remain otherwise healthy. We performed WGS on individuals from a single family to identify the genetic variant(s) segregating with the trait. The resulting sequence was compared to a recent catalog of 91 million single nucleotide polymorphisms (SNPs) and insertion/deletions (indels) identified from 144 modern breeds, together with a set of wild canids and village dogs (Plassais *et al.* 2019). Our analysis identified the same single, high-impact variant within the coding sequence of the serum/glucocorticoid regulated kinase family member 3 (*SGK3)* gene as highlighted by Hytonen and Lohi (Hytönen and Lohi 2019). We have previously shown that a distinct mutation in the same gene was responsible for hairlessness in the unrelated American Hairless Terrier breed (AHT) (Parker *et al.* 2017b). Our findings thus validate recently published findings and highlight further the importance of *SGK3* in controlling the canine hairless trait.

## Material and Methods

**Figure 1:**
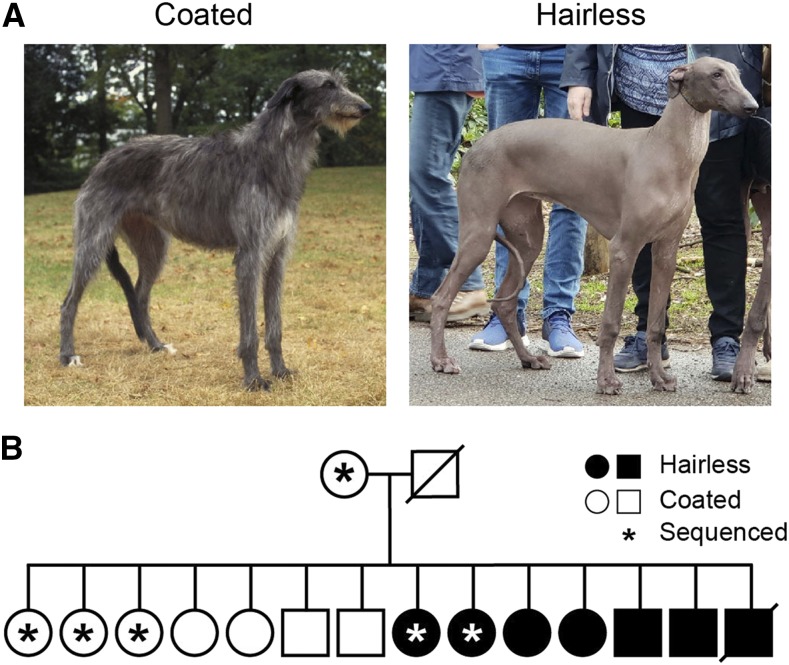
Hairless Scottish Deerhound phenotype and pedigree. (A) Typical coated and hairless Scottish Deerhounds. (B) Pedigree analyzed for this study included sire and dam, both of whom have normal coats, and five offspring, three of whom had normal coats (open symbols) with two that were hairless (filled symbols). Coated deerhound image courtesy of Mary Bloom, copyright AKC. Hairless deerhound image provided by Marjan Hemminga.

The pedigree analyzed here was composed of the sire, dam and 14 offspring. Six individuals underwent WGS: three offspring with normal coats, two hairless offspring; and the dam (Figure 1). All procedures were reviewed and approved by the National Human Genome Research Institute (NHGRI) Animal Care and Use Committee at the National Institutes of Health. Whole blood samples from the six dogs were collected into acid citrate dextrose (ACD) anticoagulant tubes. DNA extraction was performed using cell lysis followed by phenol chloroform extraction using previously published protocols (Bell *et al.* 1982).

### Whole genome sequencing and variant calling

WGS from one dam and five offspring was carried out at the NIH Intramural Sequencing Center using the Illumina TruSeq DNA PCR-Free Protocol (Cat.# FC-121-3001) on an Illumina Novaseq6000 platform. Sequence data from the six pedigree dogs is available on SRA (BioProject PRJNA576632). Dual-indexed adapters were utilized to minimize barcode switching. Paired-end data were aligned to the CanFam3.1 reference genome (http://genome.ucsc.edu/cgi-bin/hgGateway?db=canFam3) using the BWA 0.7.17 MEM algorithm (Li and Durbin 2009), sorted with SAMtools (Li *et al.* 2009), and screened for putative duplicate reads with PicardTools 2.9.2 (https://github.com/broadinstitute/picard). Sequences were locally realigned based on documented and novel insertions-deletions (Axelsson *et al.* 2013) using GATK 4.0.8.1 (DePristo *et al.* 2011), and training sets of dbSNP and Illumina Canine HD chip positions were used for base quality recalibration. HaplotypeCaller was used in gVCF mode (Poplin *et al.* 2017)to call SNVs for each individual dog, and then jointly across all dogs. The vcf file was compared to the published variant file of 91 million canine SNPs and small indels (Plassais *et al.* 2019) (https://www.ncbi.nlm.nih.gov/bioproject/PRJNA448733) using vcftools 0.1.15 –gzdiff –diff-site to retrieve all novel variants. Variants were annotated using SNPeff (Cingolani *et al.* 2012) with the CanFam3.1.86 ensemble gene list. All variants with moderate to high impact predictions were retained for further analysis. These were then filtered for the following inheritance pattern: heterozygous in the dam, homozygous alternate in the two hairless pups, heterozygous or homozygous reference in the three coated pups.

### RNA extraction and quantitative PCR

Hair was not available for nucleic acid isolation so we utilized blood as a proxy, as has been done in mice (Zetoune *et al.*, 2008). It also expresses *SGK3* and is readily available. A total of 2.5 ml of peripheral blood was collected into PaxGene Blood RNA tubes (BD Bioscience) for RNA isolation from five coated and two hairless dogs. RNA was isolated from blood using the PaxGene Blood RNA kit following manufacturer’s recommendations. Complementary DNA (cDNA) was generated using SuperScript II (ThermoFisher) using standard protocols, with 1μg of total RNA input. Quantitative PCR (qPCR) was performed using the *Power*SYBR Green PCR Master Mix (Applied Biosystems) on the CFX384 Real-Time System (Bio-Rad). Normalized *SGK3* expression was obtained by subtracting housekeeping gene (*GAPDH*, *HPTR1*, *RPS19*) threshold cycle values from those of *SGK3*. Statistical testing was not carried out due to limited sample availability. Two pairs of qPCR primers were created to ensure reproducibility of the *SGK3* gene:

*SGK3* set 1: 5′-GGGACACCAGAGTACCTTGC and 5′-GGGAGGCAATCCATACAGCA

*SGK3* set 2: 5′-CCTAATGTGGCAGGACCAGA and 5′-GCCTCCAGTACACTGGCATT

In addition, three independent control primer pairs were used as a reference set:

*GAPDH*: 5′-CCTCATGACCACCGTCCA and 5′-AAGCAGGGATGATGTTCTGG;

*HPTR1*: 5′-TTTGCTGACCTGCTGGATTAT and 5′-CCTTTCCAGTTAAAGTTGAGAGAT;

and *RPS19*: 5′-TCACTGGTGAGAACCCCCT and 5′-CCTGATTCACACGGCGTAG

### Data availability

The authors affirm that all data necessary for confirming the conclusions of the article are present within the article, figures, and tables. Data has been loaded to SRA (BioProject PRJNA576632). VCF files for the 722 control WGS are available at: (https://www.ncbi.nlm.nih.gov/bioproject/PRJNA448733). Supplemental material available at figshare: https://doi.org/10.25387/g3.10301855.

## Results

### Hairless Scottish Deerhound phenotype

The American Kennel Club (AKC) breed standard for the Scottish Deerhound describes the coat as “harsh and wiry about three or four inches long,” as seen in Figure 1A (American Kennel Club 2017). Hairless Deerhounds are extremely rare, yet we identified a family with a mix of coated and hairless offspring (Figure 1B). Both sire and dam were coated. At birth, puppies displayed either a normal, full coat or a sparse and receding coat, with the latter giving the appearance of balding dogs. In those who initially had hair but went bald, the coat progressively thinned early in life and was completely gone by five weeks (Figure 1A). Beyond the coat, no other obvious phenotypes were observed and the dogs experience no unusual health issues.

### Candidate mutation analysis

As both parents were coated and the offspring had a mix of phenotypes, a Mendelian recessive pattern of inheritance was predicted. This is reminiscent of the hairless variety of the AHT, an AKC-recognized breed whose 2004 standard calls for a completely hairless body, which is also autosomal recessive (American Kennel Club 2017). We demonstrated previously that hairlessness in the AHT was due to a recessive mutation in the *SGK3* gene: a deletion of four bases within exon four at chr29:16366702-16366705 (p.Val96GlyfsTer50). We had hypothesized that the mutation alters the reading frame of the protein, creating a new protein sequence for 50 amino acids and a premature stop at amino acid 157 which shortens the protein by 2/3. We therefore initially genotyped the mother and five Deerhound offspring for the same mutation, but observed that all dogs contained the wild type sequence at this position. This suggested to us that a unique genomic variant is responsible for hairlessness in the Scottish Deerhound.

### WGS and variant filtering

**Figure 2:**
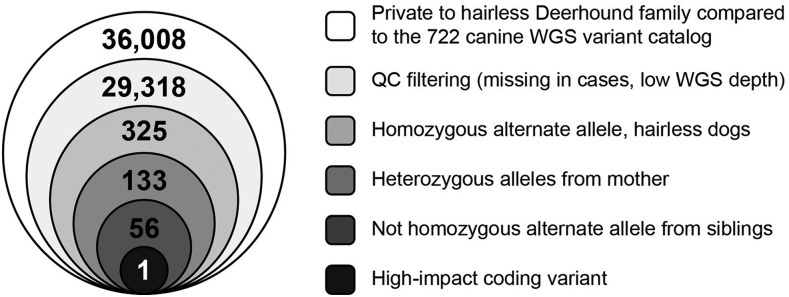
Filtering paradigm to isolate novel hairless variants. WGS data from the hairless Scottish Deerhound pedigree was compared to a recently published comprehensive catalog of 91 million SNPs and indels to identify 36,008 variants that were unique to the pedigree. Filtering based on Mendelian inheritance patterns, revealed 56 candidate variants. Only a single variant was predicted to be both within the coding region and to create a high-impact functional change.

To identify potential causative variants, we performed WGS of the dam and five offspring. We then applied a series of filters to reduce the number of likely causative variants (Figure 2). First, we subtracted all variants found within a recently published canine WGS catalog that included one Scottish Deerhound (Plassais *et al.* 2019), identifying 36,008 SNPs and indels that were private to this family. After removal of poor-quality variants due to low sequencing depth (<20 reads), missing genotypes for one hairless dog or greater than two pedigree dogs, a total of 29,318 variants remained. Filtering for the expectation of homozygosity of the alternate, non-reference allele in the hairless offspring left 325 variants. As we predict a Mendelian recessive pattern of inheritance, both parents should carry the variant allele; 325 variants were reduced to 133 by selection of variants that were heterozygous in the dam. Finally, this number was reduced to 56 by excluding variants that were homozygous alternate (homozygous for the rare allele) in the coated offspring (Supplemental Material, Table 1). Only a single variant of the remaining 56 was within an exon of a gene and is predicted to be of high-impact by SNPeff (Cingolani *et al.* 2012). This variant is a one base pair insertion in exon 2 of *SGK3* (c.137_138insT) which is predicted to cause a frameshift p.(Glu47GlyfsTer3), leading to an early termination of the protein, thus producing only 10% of its predicted 490 amino acids.

### Gene expression

**Figure 3:**
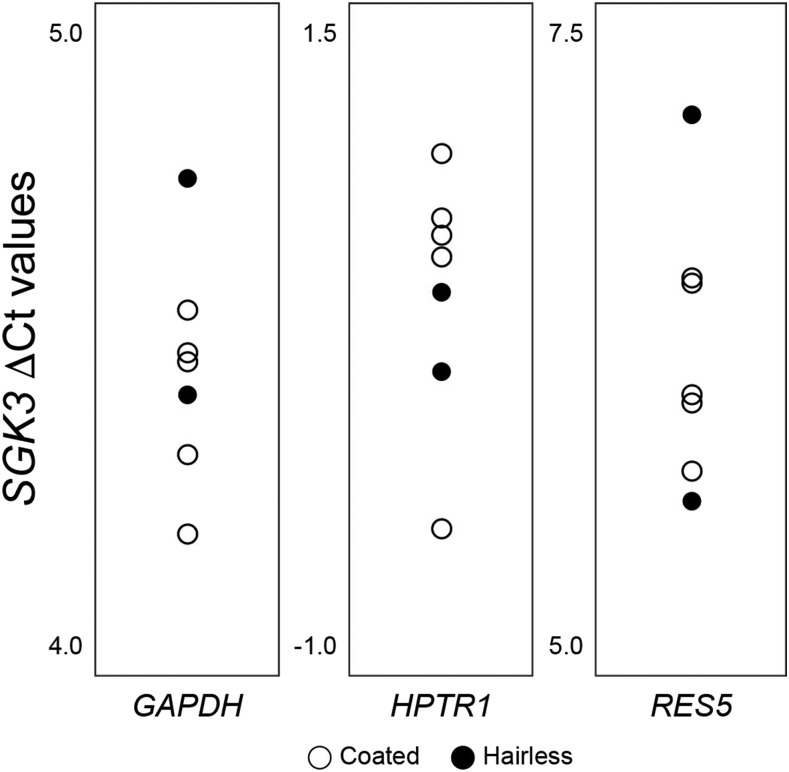
Quantitative PCR of coated and hairless Deerhound blood. Quantitative PCR against *SGK3* and housekeeping gene transcripts in both coated (open circles) and hairless (filled circles) Deerhounds have comparable ∆Ct values (*SGK3* – housekeeping), suggesting comparable blood expression values and lack of nonsense mediated decay. Statistical analysis is not possible due to the lack of sufficient available hairless individuals. Higher ΔCt values represent smaller expression levels.

This *SGK3* variant leads to a frameshift in the coding sequence and was predicted by Hytonen and Lohi (Hytönen and Lohi 2019) to lead to nonsense mediated decay. The lack of health issues in hairless Scottish Deerhounds, as opposed to observations in loss-of-function mouse models, suggests that perhaps *SGK3* is still expressed in hairless dogs. We therefore assayed gene expression levels in both coated and hairless Deerhounds. RNA was isolated from peripheral blood collected from five coated and two hairless Deerhounds to determine if *SGK3* transcript levels were reduced in hairless dogs as compared to their coated counterparts. Quantitative PCR suggests little variation between the two, although statistical analysis is not possible due to limited numbers of hairless samples available (Figure 3). The absolute cycle number for detection by qPCR does not indicate complete loss of expression, as might be expected with nonsense mediated decay. These data suggest that alternative mechanisms may lead to hairlessness in these Scottish Deerhounds but more data are needed for future investigation.

## Discussion

The domestic dog continues to be a resource for identifying variants and genes controlling a wide spectrum of morphologic features, including phenotypes related to hair, such as length, texture, curl, shedding, growth patterns, etc. Total hairlessness, or *alopecia universalis*, is a topic of interest for dog fanciers and breeders as it is typically among the most visible and, outside of a small number of selected breeds, least desirable traits. Only two genes have been associated with hairlessness in dogs. The first, *FOXI3*, is responsible for hairlessness in the Chinese Crested, Xoloitzcuintli, and Peruvian Inca Orchid dog (Drögemüller *et al.* 2008). The second, *SGK3*, was originally described by us as responsible for the recessive hairless phenotype in the AHT (Parker *et al.* 2017b). *SGK3* is a relative of the *Akt* gene and is essential for the development and maintenance of the hair follicle. Hair follicles in mice who lack *Sgk3* fail to mature normally, *e.g.*, fur proliferation is reduced and apoptosis is increased, leading to early regression of hair follicles (Alonso *et al.* 2005). Importantly, loss-of-function mouse models also display a range of other symptoms including decreased bone density, kidney stones (Bhandaru *et al.* 2011), behavioral abnormalities (Lang *et al.* 2006), and decreased intestinal glucose transport leading to delayed growth (Sandu *et al.* 2005). In contrast, neither the Deerhounds studied here nor the AHT are known to have additional gross phenotypes beyond coat aberration. Our expression analysis suggests at least partial retention of gene levels in a peripheral cell type as a proxy for intrinsic gene regulation.

While this study was ongoing, an analysis of eight normal and two hairless Scottish Deerhounds utilized homozygosity mapping to identify two probable variants within regions of shared allelic homozygosity on chromosome 29 (Hytönen and Lohi 2019), the most likely of which was the same insertion and subsequent frameshift in *SGK3* described here. We are thus able validate these results in a fully independent data set.

While the above is a likely explanation for the hairlessness phenotype, a second hypothesis is suggested by consideration of alternative transcripts. In our original analysis of the AHT we hypothesized that as a result of the four base pair deletion the entire STKc_SGK3 catalytic domain is lost. However, examination of the transcripts in both the AHT and hairless Deerhound transcripts reveals a second hypothesis of exon skipping and/or the use of a second starting methionine to produce a nearly complete protein. The predicted alternative proteins would lose most of the PX domain but retain the catalytic domain. The PX domain is required for localization of SGK3 to the endosome where activating phosphorylation occurs (Xu *et al.* 2001; Tessier and Woodgett 2006). If correct, this would explain why the hairless Scottish Deerhounds and AHT are otherwise healthy, with no deleterious phenotypes. Indeed, a protein with a functioning but inactive catalytic domain may retain reduced ability to function in other, non-hair follicle related pathways, resulting in a mild phenotype in dogs rather than the knock-out phenotype observed in mice, although we were unable to test this hypothesis.

As canine whole genome data continue to expand, it is likely that sequencing a limited number of individuals will be sufficient to identify putative causative variants. The current dataset is sufficiently large that even if we had only sequenced the two hairless dogs, we would have been able to filter our dataset to just four high-impact coding variants. Sequencing of large numbers of dogs from a maximal number of breeds, as proposed by the international Dog10K project (Ostrander *et al.* 2019), will permit identification of single associated variants across phenotpyes with the same minimal sequencing. It will also permit more rapid identification of causative variants within regulatory regions, as is expected for many diseases. The domestic dog, therefore, continues to build its reputation as a resource for understanding the genetics of traits important to human health.
